# Prediction of quantitative phenotypes based on genetic networks: a case study in yeast sporulation

**DOI:** 10.1186/1752-0509-4-128

**Published:** 2010-09-10

**Authors:** Li Shen, Iouri Chepelev, Jie Liu, Wei Wang

**Affiliations:** 1 Department of Chemistry and Biochemistry, University of California, San Diego, 9500 Gilman Drive, San Diego, CA 92093, USA; 2 Department of Neuroscience, Mount Sinai School of Medicine, 1425 Madison Avenue, New York, NY 10029, USA; 3 Laboratory of Molecular Immunology, National Institutes of Health, 9000 Rockville Pike, Bethesda, MD 20892, USA

## Abstract

**Background:**

An exciting application of genetic network is to predict phenotypic consequences for environmental cues or genetic perturbations. However, *de novo *prediction for quantitative phenotypes based on network topology is always a challenging task.

**Results:**

Using yeast sporulation as a model system, we have assembled a genetic network from literature and exploited Boolean network to predict sporulation efficiency change upon deleting individual genes. We observe that predictions based on the curated network correlate well with the experimentally measured values. In addition, computational analysis reveals the robustness and hysteresis of the yeast sporulation network and uncovers several patterns of sporulation efficiency change caused by double gene deletion. These discoveries may guide future investigation of underlying mechanisms. We have also shown that a hybridized genetic network reconstructed from both temporal microarray data and literature is able to achieve a satisfactory prediction accuracy of the same quantitative phenotypes.

**Conclusions:**

This case study illustrates the value of predicting quantitative phenotypes based on genetic network and provides a generic approach.

## Background

Predicting the consequences of environmental cues or genetic perturbations based on genetic network is becoming a powerful tool to understand biological phenomena or gene functions from a systems point of view. Ordinary differentiation equation (ODE) can make detailed predictions on a network but its application is limited by the network size because determining the values of the kinetic parameters for a large number of ODEs and solving these questions are often nontrivial. Recently, one exceptional study was conducted on an archaeon *H. salinarum NRC-1 *[1]. Subsequent to genome sequencing, a large number of microarray, proteomic and ChIP-chip assays were carried out to reconstruct the genetic network. The great amount of data allowed training of a computational method to predict expression changes of gene modules upon perturbations. Ideally, such a comprehensive and systematic approach can be applied to every organism. However, the tremendous expense and effort are often inhibitory particularly for much more complicated organisms such as human. Alternatively, large-scale networks have been reconstructed from genomic and proteomic data. Although relatively noisier than the genetic networks studied by ODE, which are usually assembled from literature, such large-scale networks can still generate insightful predictions. For example, Marcotte and colleagues have predicted the phenotypes of knocking out genes in yeast and worm using genetic networks reconstructed by integrating various sources of data [2,3]. However, these networks only represent correlation between genes but not necessarily physical interactions. Predictions are made based on how tightly the gene of interest is correlated with genes annotated with desired specific phenotype. Similar approaches have been applied to predicting gene functions, particularly those related to diseases and thus potential drug targets, based on networks directly reconstructed from genomic and/or proteomic data [4-10].

In the present study, we aim to address the following issues in predicting phenotypes based on genetic network. First, can one perform *de novo *predictions of phenotypes without relying on existing annotations of genes? If this is feasible, it will not only help make new discoveries but also demonstrate the effectiveness of understanding biological phenomena at a systems level. Second, can one predict a phenotype that is quantitatively measured using a genetic network that consists of physical interactions? A quantitative phenotype may provide a rigorous assessment of the prediction accuracy and physical-interaction network often shed light on understanding the molecular mechanism of phenotype formation. Third, can genomic analysis capture the most prominent features, which may form the major regulatory interactions, of such network? Is this "scaffold" of the network still able to predict the quantitative phenotypes?

We choose the sporulation process in *Saccharomyces cerevisiae *to perform a case study. All sexually reproducing organisms undergo meiosis in which each diploid cell generates four haploid gametes. The meiotic process in budding yeast is coupled with spore morphogenesis in which spores are formed from the haploid cells. Regulation of yeast sporulation has been studied for years and numerous important regulators have been identified [11,12]. Genome-wide expression assays have been performed to determine the transcriptional program [13-15]. In addition, effect of single-gene deletions on sporulation efficiency has been determined at a genomic scale, which provided quantitative phenotypic measurements [16].

We first collect experimental evidence from literature to construct a network that includes both protein-protein interactions and transcription factor (TF)-gene regulatory interactions. We then investigate the dynamics of the network using a Boolean network model. Our study demonstrates that the yeast sporulation network has a robust design and, once sporulation starts, the network topology ensures the completion of the process. We also reconstruct a transcriptional regulatory network for yeast sporulation from genomic data using a computational method called UMMI (Ubiquitous Model selector for Motif Interactions). Comparison between the curated and the predicted networks shows that the most important transcriptional edges of the curated network are correctly identified by UMMI. When the predicted transcriptional edges are combined with necessary non-transcriptional edges taken from literature, the hybrid network shows the same dynamic characteristics and similar predictive power as the fully curated one.

## Results

### Construct yeast sporulation network from literature

**Figure 1:**
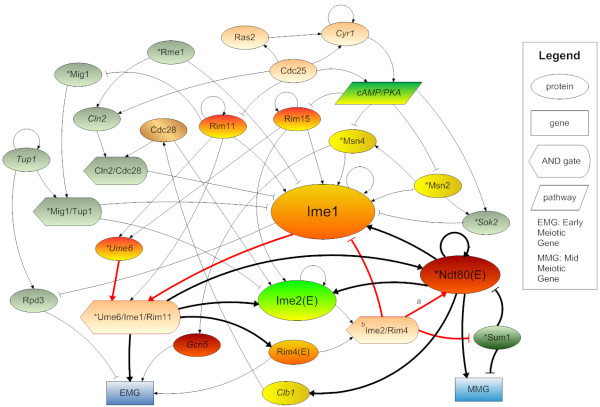
**The yeast sporulation network constructed from literature**. The bold links are regulatory interactions found by UMMI. Ime2, Rim4, and Ndt80 are early meiotic genes and are thus denoted with an "E" in parentheses. They are repressed by Rpd3 and activated by Gcn5 like other EMGs. These edges are not shown for the sake of clarity. The DNA-binding proteins are denoted with an asterisk before their names. ^a ^The phosphorylation of Ime2 to Ndt80 is still hypothetical. However, removal of this edge does not affect our results (see Additional file 1). ^b ^Rim4 acts as an RNA binding protein to stabilize Ime2 mRNA (see Additional file 1).

We first construct a genetic network with 29 nodes for yeast sporulation from the literature [11,17]. This curated network includes the known major regulators for the yeast sporulation such as Ume6, Ime1, Sum1 and Ndt80 (Fig. 1). Completion of the sporulation process requires sequential activation of the early meiotic genes (EMG) and the middle meiotic genes (MMG), which are represented by two marker nodes in Fig. 1. We use AND nodes to represent the formation of protein complexes (see Methods). The cAMP/PKA signaling pathway plays an important role in yeast cells to prevent sporulation under growth condition [11]. It suppresses the activity of several major sporulation activators such as Rim15 and Msn2. Therefore, we introduce a single suppressor node to represent this pathway.

The upper half of Fig. 1 shows that many protein-protein interactions are involved in regulating a master meiotic regulator - Ime1. After Ime1 is activated, it turns on the downstream sporulation activators such as Ime2 and Ndt80 to transcribe EMG and MMG (the lower half of Fig. 1). After both EMG and MMG are transcribed, the yeast cell is committed to complete the sporulation process [15].

### Predict the yeast sporulation efficiency

A genome-wide study was performed previously to quantitatively determine the effect of deleting a single gene on the efficiency of yeast sporulation [16]. A Prespo/Spore ratio, measured by microarray, represents the percentage of a single deletion strain that can complete sporulation. If the ratio is larger than one, the deleted gene is considered as sporulation deficient; otherwise, sporulation efficient.

We choose Boolean network to analyze the curated network and search for the fixed points (Fig. 1). We follow the previous work of [18] in updating the network state (defined as the states of all nodes in the network) using a Markov chain (see Methods). The only modification to the previous method is the inclusion of a logical AND function to mimic the effect of an AND node. We also define a product function to quantify the sporulation percentage using the two markers' states: when EMG and MMG are both in state "1", sporulation is complete; otherwise, sporulation is incomplete. Perturbations to the network can be performed by clamping a node to state "0", for deleting a gene, or removing an edge, for disrupting an interaction. To have a direct comparison with the measured Prespo/Spore ratio, we calculate the ratio of sporulation percentage before perturbation versus that after perturbation, denoted by a symbol *a *(see Methods). This is done by enumerating all possible initializations of Boolean networks with and without clamping the deleted gene to state "0". In the same way, we have also evaluated the effect of other types of perturbations to the network, such as deleting an edge or deleting multiple genes (see below).

**Figure 2:**
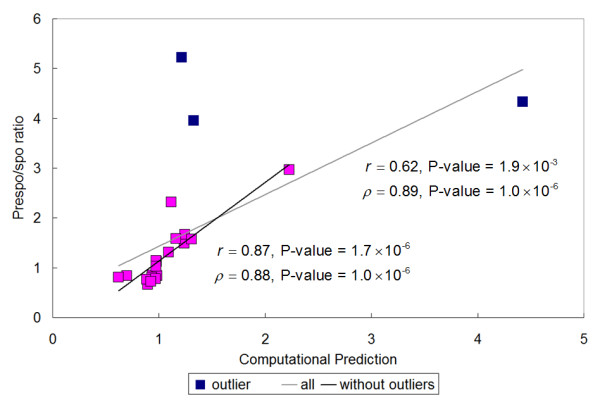
**Correlation between the Prespo/Spore ratios and the sporulation efficiency predicted using the curated network**. Two types of correlation, Pearson correlation *r *and Spearman rank correlation *ρ*, are calculated. The three outliers are colored in blue and the correlations are also calculated without the outliers.

We observe that the sporulation percentage for the curated network is 0.61 (without any perturbation). All individual nodes in Fig. 1 are then systematically deleted (clamped) except AND gate, pathway, EMG and MMG nodes that do not represent specific genes in the microarray experiments [16]. For the 22 genes deleted in the curated network (Fig. 1), satisfactory correlation between the measured and predicted sporulation efficiency are observed (Fig. 2 and Additional file 1, Table S2). The Pearson correlation is 0.62 with a P-value of 1.9 × 10^-3 ^and the Spearman rank correlation is 0.89 with a P-value of 1.0 × 10^-6^. There are three outliers in Fig. 2: Rim4, Rim11 and Ndt80. RIM4 is required for high-level gene expression in the early stage of meiosis, premeiotic DNA replication, timely and efficient commitment to meiotic recombination, nuclear division, and spore formation. Rim11 is a protein kinase required for the interaction between Ime1 and Ume6, and subsequently the expression of EMG and spore formation. Rim11 is also required to relieve the repression of Ime1. However, the exact mechanisms of Rim4 and Rim11 to promote sporulation are still unknown. It is most likely that there are regulatory partners of Rim4 and Rim11 missed in the curated network. Also, the simple Boolean network may not be able to capture sophisticated regulatory interactions, such as the competitive regulation between Ndt80 and Sum1 [19]. Nevertheless, the high Spearman rank correlation shows that our model correctly captures the relative effect of single gene deletions. The third outlier Ndt80 is a very important meiotic regulator and its deletion is highly sporulation deficient (Prespo/Spore ratio = 4.33). Our prediction (*a *= 4.42) corresponds very well with the experimental result. We consider it as an outlier to avoid the correlation result biased by a single data point. If the three outliers are removed, the Pearson correlation becomes 0.87 with a P-value of 1.7 × 10^-6 ^and the Spearman rank correlation becomes 0.88 with a P-value of 1.0 × 10^-6^, which are satisfactory considering the difficulty of *de novo *prediction on quantitative phenotypes.

To further illustrate obtaining such a correlation is nontrivial, we perform a negative control experiment by looking at the correlation between the "absolute" sporulation efficiency change caused by deleting a gene and the averaged or minimal shortest path from each gene to EMG and MMG. To calculate the "absolute" sporualtion efficiency change, Prespo/Spore ratios smaller than 1.0 are inversed. A negative correlation is thus expected because the shorter a gene's path to the markers, the larger its influence. If the averaged path is used, we have a Pearson correlation of -0.45 with a P-value of 0.04 and a Spearman rank correlation of -0.53 with a P-value of 0.01. If the minimal path is used, a Pearson correlation of -0.45 with a P-value of 0.03 and a Spearman rank correlation of -0.49 with a P-value of 0.02 are obtained. Both the correlation and the statistical significance are significantly lower than Boolean network predictions.

### Robustness and hysteresis of the sporulation network

The satisfactory performance of the curated network in predicting sporulation efficiency for single-gene deletions suggests that this network captures the major regulatory interactions of yeast sporulation. We thus further analyze this network (Fig. 1) to investigate how robust the yeast sporulation process is. Once Ime1 is activated, the cell is considered to commit to meiosis. The three key regulators (Ime1, Ime2 and Ndt80) have multiple positive feedbacks to sustain their active status (Table 1 and Fig. 1). Therefore, we first examine which of these feedbacks are important for sporulation completion. We disrupt each feedback loop by removing the edge in the Boolean network and re-calculate the sporulation percentage by enumerating all possible initializations (Table 2). None of the perturbations causes significant change in sporulation percentage except Ime2--|Sum1 (1.59), which confirms the importance of relieving the repression of Sum1 on NDT80. We then perform a systematic study by knocking out every edge in the curated network and analyze their effects on sporulation efficiency (Additional file 2, Table S11). For those edges whose deletions are sporulation deficient, only Ndt80-> MMG (3.89) and Ime1--| Rpd3 (1.30) (besides Ime2--|Sum1) have high and intermediate impact on sporulation, respectively. The former is obvious because Ndt80 is a key activator for MMG while the latter shows the importance for Ime1 to repress an EMG repressor, Rpd3. The other 41 edges only affect sporulation efficiency slightly (Additional file 2, Table S11). The deletion of the rest 26 edges is sporulation efficient but only five of them have relatively significant effect (*a *≤ 0.75). Notably, four of them are related to an EMG repressor, Rpd3; Rpd3--|Rim4 (0.67); Rpd3--|EMG (0.68); Tup1-> Rpd3 (0.69) and Tup1-> Tup1 (0.75). Tup1 is also a repressor for Ime1. The fifth sporulation efficient edge is cAMP/PKA--|Msn2 which belongs to the cAMP/PKA pathway. All of these sporulation efficient edges are related to pre-meiotic repression of EMG and IME1, which is important to prevent sporulation under the growth condition. Our analysis suggests that the sporulation network is overall robust, which guarantees the completion of sporulation once the cell is committed to it.

**Table 1:** Positive and negative feedback loops of the three regulators of sporulation*.

Regulator	Function	Feedback loops	
Ime1	P	i.	Ime1-> Ime1;
		ii.	Ime1->Ndt80-> Ime1;
		iii.	Ime1->Ime2->Ndt80-> Ime1.

Ime2	P	i.	Ime2-> Ime2;
		ii.	Ime2->Ndt80-> Ime2;
		iii.	Ime2--|Sum1--|Ndt80-> Ime2;
		iv.	Ime2->Ndt80->Clb1->Cdc28-> Ime2.

Ndt80	P	i.	Ndt80-> Ndt80;
		ii.	Ndt80->Ime2-> Ndt80;
		iii.	Ndt80-> Ime2--|Sum1--|Ndt80;
		iv.	Ndt80->Ime1-> Ndt80;
		v.	Ndt80->Clb1->Cdc28->Ime2-> Ndt80.

Ime1	N	i.	Ime1-> Ime2/Rim4--|Ime1;
		ii.	Ime1->Ndt80->Clb1-> Cln2/Cdc28--|Ime1.

* "-> " means the left node activates the right node and "--|" means the left node represses the right node. "P" and "N" represent that the functions of the feedback loops are positive and negative regulation, respectively.

**Table 2:** Effects of removing positive feedback loops.

Perturbation	a*
Ime1 auto-regulation	1.11

Ime1-> Ndt80	1.04

Ndt80-> Ime1	1.14

Ime1-> Ime2	1.00

Ime2-> Ndt80	1.08

Ime2 auto-regulation	1.00

Ndt80-> Ime2	1.00

Ime2--|Sum1	1.59

Sum1--|Ndt80	0.98

Ndt80-> Clb1	0.99

Clb1-> Cdc28	0.97

Cdc28-> Ime2	1.00

Ndt80 auto-regulation	1.01

*a represents the effect of disrupting the edge to the completion of sporulation (see definition of *a *in the text).

In addition to positive feedbacks, there are two negative feedback loops for Ime1 (Table 1). Such architecture determines that Ime1 forms a hysteretic switch of sporulation: Ime1 is absolutely needed to initiate the meiotic process; however, Ime1 becomes unneeded after the cell commits to sporulation. Indeed, it is known to be important for the yeast cell to inactivate Ime1 once the sporulation-specific genes have been transcribed [20]. Consistently, we observe that Ime1 is in the final state "1" in only 44% of all possible initializations that lead to sporulation in the curated network, indicating the importance of the negative feedbacks. To further confirm this, we perturb the two negative feedback loops (Table 1) by deleting the repression edges to Ime1. Removing either Cln2/Cdc28--|Ime1 or Ime2/Rim4--|Ime1 raises the percentage to 61% in both cases. Removing both edges raises the percentage to 70%.

### Predictions of other perturbations' effects on sporulation

**Figure 3:**
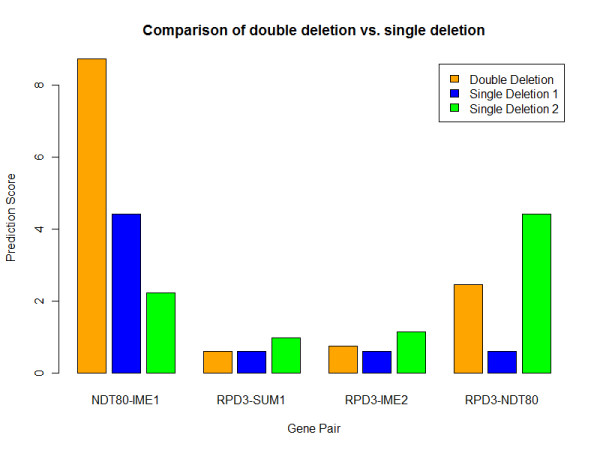
**Comparison of prediction scores of double deletion vs. single deletion**. The bars in orange color are double deletion scores. The bars in blue and green colors are single deletion scores for the left and right genes in the gene pairs, respectively.

To identify synergetic genetic interactions between genes, we perform double deletion experiments in the curated network to identify gene pairs that are either sporulation deficient or efficient. From the histogram of the 231 double deletion tests (Additional file 1, Fig. S1), four groups of gene pairs emerge: sporulation efficient (*a *≤ 0.90, 19.1%); sporulation neutral (0.9 <*a *≤ 1.57, 59.7%); middle sporulation deficient (1.57 <*a *≤ 4.00, 14.7%) and high sporulation deficient (*a *> 4.00, 6.5%). These thresholds are consistent with the Prespo/Spore ratios used in [16] to define sporulation-deficient and -efficient genes. The full list of gene pairs and deletion results are shown in Additional file 3, Table S9). The most sporulation deficient gene pair is Ndt80-Ime1 (*a *= 8.72) compared to *a *= 4.42 and *a *= 2.23 for Ndt80 and Ime1 single deletions, respectively (Fig. 3). This is not surprising because they are master regulators for early and middle meiotic genes. The most sporulation efficient gene pair is Rpd3-Sum1 (*a *= 0.62) (Fig. 3). Histone deacetylase Rpd3 is an early meiotic repressor [21]. Seventeen gene pairs associated with Rpd3 are sporulation efficient (*a *varies from 0.62 to 0.64). However, association with other regulators may alleviate the effect of Rpd3 deletion, e.g. Rpd3-Ime2 (*a *= 0.77) and Rpd3-Ndt80 (*a *= 2.47) (Fig. 3). Together with the feedback loop deletion analysis, our study manifests the importance of Rpd3 in regulating sporulation progression and completion.

We also find that the self-activation of the meiotic activators has minor impact on sporulation efficiency (Additional file 1, Table S4). Even when the self-activation of all five activators is disrupted, the effect is slightly sporulation deficient (*a *= 1.27). However, the PKA pathway plays an important role in suppressing sporulation as deletion of cAMP/PKA node is sporulation efficient (*a *= 0.74). This is consistent with the known role of this pathway in the literature [11]. All these computational predictions are novel and can guide the future experimental investigations of the sporulation mechanisms.

### Uncover transcriptional regulatory interactions of sporulation by a computational method

We finally exploit a computational approach, UMMI, for *de novo *discovery of the transcriptional regulatory interactions during the sporulation. UMMI is an extension to our previous method, GBNet [5], which aims to find sequence constraints, such as co-occurrence of two motifs and distance constraint between them, enriched in a group of co-regulated genes. Based on the rules identified, target genes of a TF can be inferred. Unlike GBNet that relies on gene clusters generated from multiple microarray experiments, UMMI can be applicable to a single gene expression experiment. In addition, we also develop a measurement in UMMI to control the reliability of the models discovered (see Methods). The gene expression data from Chu et al. [13] is used in our analysis, which covers seven time points of sporulation: Metabolic (0 h), Early I (0.5 h), Early II (2 h), Early-Mid (5 h), Middle (7 h), Mid-Late (9 h) and Late (11.5 h).

We have compiled a list of 794 DNA motifs in yeast, including known motifs taken from literature and computationally generated ones (see Additional file 1). At each time point, all the genes are divided into 5 groups based on their expression levels (see Methods). UMMI is then used to find the combination of motifs and sequence constraints between these motifs that are associated with gene expression levels. UMMI finds several highly reliable constraints at each time point (Additional file 1, Table S1) that pass a reliability threshold (frequency of occurrence in the learned models) of 0.1. Based on these sequence constraints, we have recovered the known key transcription factors (TFs) in sporulation: Ume6, Ndt80 and Sum1. Furthermore, we identify 75 Ume6's target genes that satisfy the Ume6's sequence constraints and show at least 2-fold over-expression at early stages of sporulation (0.5-5 h). The functions of these genes indicate that they play important roles in sporulation. For example, the top three enriched gene ontology (GO) terms of biological process are: M phase of meiotic cell cycle (3.1E-18), meiosis I (2.2E-17) and reciprocal meiotic recombination (1.3E-14) (See Additional file 4, Table S5). We also identify 263 and 121 target genes whose expression levels have at least 2-fold elevation at middle stages (5-9 h) and satisfy the sequence constraints for Ndt80 and Sum1, respectively. The top three enriched GO terms of biological process are: spore wall assembly (4.5E-17 and 3.5E-21), sporulation (4.4E-15 and 1.0E-18) and ascospore formation (1.8E-14 and 1.3E-18) (P-values for Ndt80 and Sum1, respectively) (See Additional file 5, Table S6 and Additional file 6, Table S7). Ndt80 and Sum1 share 49 common targets whose top three enriched GO terms for biological process are strongly associated with sporulation: spore wall assembly (1.1E-16), sporulation (3.2E-16) and ascospore formation (1.3E-15) (See Additional file 7, Table S8 for full list).

**Figure 4:**
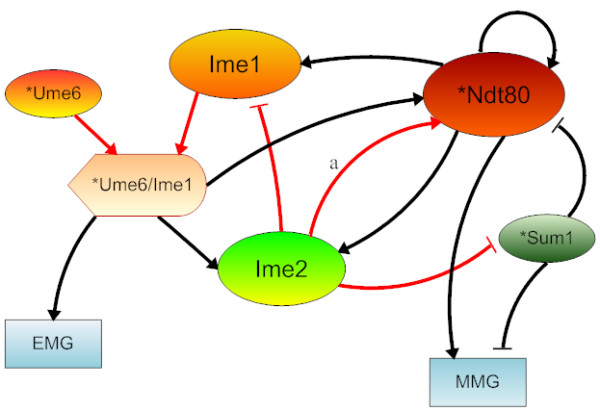
**The yeast sporulation network consisting of the core transcriptional regulations identified by UMMI from microarray experiments**. The type of the black links is determined by literature knowledge. The red links are essential protein-protein interactions taken from the literature. ^a ^See legend of Fig. 1.

We compare the target genes of the three regulators to the known regulators of sporulation [11,17] and, based on the overlapped targets (Table 3), we reconstruct a sporulation network of the core transcriptional regulations with minimal protein-protein interactions (PPI) added from literature (Fig. 4). The added PPI are: a complex formation between Ume6 and Ime1; Ime2's repression on Ime1 and Sum1 by phosphorylation; Ime2's activating Ndt80. These PPIs are essential to sporulation but cannot be detected by gene expression microarray experiments. They are thus added to complete the network in Fig. 4. We also add an Ume6/Ime1 node to represent the protein complex formed by Ume6 and Ime1. We denote the Ume6's target genes as "EMG", and Ndt80 and Sum1's target genes as "MMG". Fig. 4 illustrates a scaffold of the genetic network of the yeast sporulation. It is no doubt many regulatory interactions are not included in this predicted network. However, the significant overlap between the curated and the predicted networks (Fig. 1 and 4) suggests that UMMI uncovers the most prominent features of a transcriptional network, which may constitute the scaffold of the genetic network regulating sporulation.

**Table 3:** Known sporulation regulators as targets of Ume6, Ndt80 and Sum1.

TF	Targets
Ume6	RIM4, IME2, NDT80

Ndt80	CLB1, NDT80, IME2, IME1

Sum1	NDT80

Interestingly, the predicted network is able to achieve comparable accuracy on sporulation efficiency prediction as the curated network (Additional file 1, Table S3). The Pearson correlation between the computational prediction and the experimental measurement is 0.87 with a P-value of 5.8E-2 and the Spearman rank correlation is 0.67 with a P-value of 0.27. It should be noted that the dataset used to calculate the correlations for the predicted network is very small (five data points) and therefore the P-values are not highly significant. Nevertheless, these encouraging results suggest that the most prominent transcriptional regulatory interactions captured by genomic data can be recovered by computational methods combined with literature curation and such a hybrid network still has a satisfactory predictive power of phenotypes.

## Discussion and Conclusions

Accurately predicting phenotypes based on genetic network that constitutes physical interactions can provide great mechanistic insight into phenotype formation. We have conducted a case study in yeast sporulation by predicting a quantitative phenotype, sporulation efficiency change after deleting a gene, based on a network assembled from the existing knowledge. Such a physical interaction network illustrates how the perturbations are propagated in the network to cause phenotype formation. Importantly, our predictions are *de novo *and only rely on network topology. This study is the first to reveal the direct relationship between network topology and phenotype formation.

It is no doubt that there are genes and/or links missing in the reconstructed network which is still small. However, the satisfactory prediction accuracy suggests that the major regulatory interactions have been uncovered. We have also demonstrated that computational methods can extract the most prominent features of the transcriptional regulation captured by genomic data. With a minimal set of protein-protein interactions added, such a scaffold network shows promising predictive power. Such a network can still be noisy but may contain the key regulatory interactions that are important to correctly predict phenotypes to a satisfactory extent.

We choose Boolean network to analyze the dynamics and robustness of the yeast sporulation network. Compared with the differential equation approach, Boolean network does not provide the detailed temporal change of each gene/protein or cooperation between genes such as the competitive binding of Ndt80 and Sum1 [19]. On the other hand, it allows study of the network in Fig. 1 with 29 nodes, which is often difficult to determine all the kinetic parameters needed in differential equations and even more challenging to solve them. Encouragingly, such a simple and easy to implement model can make *de novo *predictions of phenotypes accurately. It would be interesting to explore the power of this model on much larger networks with hundreds or even thousands of nodes once the data for reconstructing the networks become available.

In this work, we predict the phenotype based on enumerating all possible initializations. The computational cost for such an approach is exponential in scale. For example, the computational time for a Boolean network with 30 nodes is 2 hours. It will be 83 days for a Boolean network with 40 nodes, and 228 years for 50 nodes. Instead of enumerating all possible initializations, we have tried to sample only a small number of the initializations at random. We find such a sampling approach can make predictions with high precisions (results not shown). Other researchers have made similar observations about Boolean networks: given the number of nodes and the node connectivity, randomly sampled Boolean networks have similar global properties [22]. We thus argue that the random sampling technique may allow extension of our approach to a much larger network in the future.

## Methods

### Boolean network

A Boolean network [23] is a graphical representation of a set of Boolean variables whose states are determined by the other variables in the network. When used to describe a gene regulatory network, the two states of a node represent the status of a protein/gene being active "1" or inactive "0" [24]. It is a simplification to use a Boolean network to describe a gene regulatory network whose nodes' states are much more complicated than on/off in reality. However, it has been shown that a Boolean network can be used to capture the global dynamics of the yeast cell cycle network [18]. In [18], the durations of the actions of genes and proteins have been reduced into a single step of updating all the nodes in the network. That is, the protein states in the next time step are determined by the protein states in the present time step by the following rule:

$$s_{i}\left(t+1\right)=\{\begin{array}{ll} 1, & \sum_{j}a_{ij}s_{j}\left(t\right)>0\\ 0, & \sum_{j}a_{ij}s_{j}\left(t\right)<0\\ s_{i}\left(t\right), & \sum_{j}a_{ij}s_{j}\left(t\right)=0 \end{array}$$

where *s_i_*(*t *+ 1) and *s_j_*(*t*) are states of node i and j at time point t and t+1, respectively; *a_ij _*= -1 if the arrow from j to i is repression "--|"; *a_ij _*= 1 if the arrow from j to i is activation "-> ". Self-degradation rule is employed to determine the states for those proteins that are not negatively regulated by others [18]. Our only modification to this method is the addition of an AND node which implements the AND logic to mimic the cooperation between proteins:

$$s_{i}\left(t+1\right)=\prod_{j}s_{ij}\left(t\right)$$

where *s_ij _*is the state of parent node j of node i at time point t.

In this work, we initialize the network with all possible states and update all nodes synchronously. All the states then evolve into a set of converged states called attractors [23]. A product function can be defined on the attractors, e.g. the percentage of node × being in state "1" among all attractors. The value of the product function therefore reflects the stable and overall dynamics of the gene regulatory network under study, which is also determined by the Boolean function and the connectivity among the nodes in the network.

We are interested in the completion of the yeast sporulation process. According to [13] (Fig. 4B), we define the product function as the percentage of EMG and MMG both in state "1":

$$f\left(x,e\right)=\frac{\sum_{A\in Attractors}I\left(A_{EMG}=1\text{ \& }A_{MMG}=1\right)}{Attractors}$$

where **x **represents nodes, **e **represents edges and *I *is the identity function (equals one if the condition is satisfied or zero otherwise). Perturbation to the network can be represented by clamping the corresponding nodes to "0" or deleting the relevant edges. For example, deletion of gene *i *corresponds to *f*(**x**_*i *= 0_, **e**) and deletion of edge between gene *i *and *j *corresponds to f(**x**,**e**_(*i*, *j*) = 0_).

In the experiments of [16], deletion strains were bar-coded by specific probes. The Prespo/Spore value for each deletion strain is the ratio of pre-sporulation probe intensity measured from a pre-sporulation culture to the spore probe intensity measured from a pure spore sample. To compare with this ratio, we calculate the effect of deleting gene *i *as:

$$a_{i}=\frac{f\left(x,e\right)}{f\left(x{|}_{i=0},e\right)}$$

The predictive power of our model can therefore be evaluated by the correlation between the values of *a *and the Prespo/Spore ratios.

### UMMI

In our previous work, we have developed a method called GBNet [5] to search for the sequence features that are enriched in a set of co-regulated genes. GBNet employs a Bayesian network to represent the grammar (regulatory rules) of cis-regulation. In the Bayesian network, a binary child node denotes a gene's category (target or background) and binary parent nodes denote the presence of DNA sequence constraints in the genes' promoters, which include motif presence, motif distance relative to TSS, spacing between two motifs, orientation of a motif, presence of a second copy of a motif and order between two motifs [5]. The objective function of the Bayesian network learning is to maximize the posterior probability of the network structure:

(1) $$\begin{array}{l} \log_{10}(P(N_{s}|D))=-N_{p}\log_{10}(K)+\\ \sum_{j=1}^{q}\log_{10}\frac{\Gamma (a_{j})}{\Gamma (a_{j}+N_{j})}\sum_{k=0}^{r}\log_{10}\frac{\Gamma (a_{jk}+N_{jk})}{\Gamma (a_{jk})} \end{array}$$

where *N_S _*is network structure, *D *is data, Γ(.) is the gamma function, N_p _is the number of parent nodes, log_10_(*K*) is a network parameter to penalize the complex models, *q *is the number of possible parent states, *r *+ 1 is the number of possible child states, $a_{j}=\sum a_{jk}$, $N_{j}=\sum N_{jk}$, *N_jk _*is the number of samples for child state *k *when parent state is *j*, *a_jk _*is a prior count. In GBNet, we only considered the case of *r *= 1, i.e. the child node is a binary variable. In order to achieve efficient structure learning, we have utilized a Gibbs sampling to search for global optimum and applied GBNet successfully to several datasets in yeast and human [5].

UMMI is an extension of GBNet with the flexibility to analyze a single microarray experiment. In UMMI, we extend the GBNet framework to consider a child node with more than two categories. That is, we allow *r *> 1 in Eq. 1. When analyzing a single microarray data, we first separate all genes into multiple categories and each category represents genes with similar expression levels (Additional file 1, Fig. S2). In this study, we choose to use five categories (*r *= 4) that span the whole spectrum of the gene expression levels with equal intervals. As in GBNet [5], the gene category labels and promoter sequences (600 bps upstream of the start codon) are fed into UMMI to learn the sequence constraints.

Each motif's ability to discriminate gene categories is evaluated by a Bayesian score which is the logarithm of the posterior probability of the Bayesian network (Eq. 1). In GBNet [5], we first rank the motifs by their Bayesian scores; a motif and its associated sequence constraints with higher rank are always tested before the motifs with lower rank [5]. To avoid data-overfitting in Bayesian network learning [5], each model learned is only allowed to have a small number of parent nodes (i.e. regulatory rules). Therefore, those highly ranked motifs may dominate the results. To avoid this possible bias, UMMI generates 51 models for each gene expression dataset (Additional file 1, Fig. S2): one model is obtained by using motifs ranked by their Bayesian scores as the input, the other 50 models by using motifs in random order as the input. This way we hope to avoid bias towards the top ranked motifs and to obtain models that may have lower Bayesian scores but are still biologically meaningful. From the 51 learned models, we calculate each sequence constraint's occurrence and only consider significantly present sequence constraints as reliable. A heuristic threshold of 0.1 for occurrence is used in this study.

## Authors' contributions

LS conceived the entire study, carried out data analysis, developed UMMI, adapted the Boolean network program for this study and wrote the paper. IC developed the Boolean network program. JL helped to collect the motif and gene expression data for UMMI to reconstruct the predicted network. WW conceived and supervised the entire study, contributed to data analysis and revised the manuscript. All authors read and approved the final manuscript.

## Supplementary Material

Additional file 1 **Supplemental materials**. Notes about UMMI and construction of the curated and predicted networks. Supplemental Tables S1-4, S10 and Figures S1-2. Click here for file

Additional file 2 **Table S11**. Effects to the sporulation efficiency by knocking out every edge in the curated network. Click here for file

Additional file 3 **Table S9**. Full list of gene pairs and double deletion results. Click here for file

Additional file 4 **Table S5**. GO terms analytic outputs of Ume6's target genes. Click here for file

Additional file 5 **Table S6**. GO terms analytic outputs of Ndt80's target genes. Click here for file

Additional file 6 **Table S7**. GO terms analytic outputs of Sum1's target genes. Click here for file

Additional file 7 **Table S8**. GO terms analytic outputs of Ndt80 and Sum1's common target genes. Click here for file

## References

[B1] Bonneau R Facciotti MT Reiss DJ Schmid AK Pan M Kaur A Thorsson V Shannon P Johnson MH Bare JC A predictive model for transcriptional control of physiology in a free living cell Cell 2007 131 7 1354 1365 10.1016/j.cell.2007.10.053 18160043

[B2] McGary KL Lee I Marcotte EM Broad network-based predictability of Saccharomyces cerevisiae gene loss-of-function phenotypes Genome Biol 2007 8 12 R258 10.1186/gb-2007-8-12-r258 18053250 PMC2246260

[B3] Lee I Lehner B Crombie C Wong W Fraser AG Marcotte EM A single gene network accurately predicts phenotypic effects of gene perturbation in Caenorhabditis elegans Nat Genet 2008 40 2 181 188 10.1038/ng.2007.70 18223650 PMC13030915

[B4] Kim WK Krumpelman C Marcotte EM Inferring mouse gene functions from genomic-scale data using a combined functional network/classification strategy Genome Biol 2008 9 Suppl 1 S5 10.1186/gb-2008-9-s1-s5 18613949 PMC2447539

[B5] Shen L Liu J Wang W GBNet: Deciphering regulatory rules in the co-regulated genes using a Gibbs sampler enhanced Bayesian network approach BMC Bioinformatics 2008 9 1 395 10.1186/1471-2105-9-395 18811979 PMC2571992

[B6] Wu X Jiang R Zhang MQ Li S Network-based global inference of human disease genes Mol Syst Biol 2008 4 189 10.1038/msb.2008.27 18463613 PMC2424293

[B7] Chen J Aronow BJ Jegga AG Disease candidate gene identification and prioritization using protein interaction networks BMC Bioinformatics 2009 10 73 10.1186/1471-2105-10-73 19245720 PMC2657789

[B8] Karni S Soreq H Sharan R A network-based method for predicting disease-causing genes J Comput Biol 2009 16 2 181 189 10.1089/cmb.2008.05TT 19193144

[B9] Huttenhower C Haley EM Hibbs MA Dumeaux V Barrett DR Coller HA Troyanskaya OG Exploring the human genome with functional maps Genome Res 2009 19 6 1093 1106 10.1101/gr.082214.108 19246570 PMC2694471

[B10] Zhu J Zhang B Smith EN Drees B Brem RB Kruglyak L Bumgarner RE Schadt EE Integrating large-scale functional genomic data to dissect the complexity of yeast regulatory networks Nat Genet 2008 40 7 854 861 10.1038/ng.167 18552845 PMC2573859

[B11] Kassir Y Adir N Boger-Nadjar E Raviv NG Rubin-Bejerano I Sagee S Shenhar G Transcriptional regulation of meiosis in budding yeast Int Rev Cytol 2003 224 111 171 10.1016/S0074-7696(05)24004-4 12722950

[B12] Vershon AK Pierce M Transcriptional regulation of meiosis in yeast Current Opinion in Cell Biology 2000 12 3 334 339 10.1016/S0955-0674(00)00104-6 10801467

[B13] Chu S DeRisi J Eisen M Mulholland J Botstein D Brown PO Herskowitz I The Transcriptional Program of Sporulation in Budding Yeast Science 1998 282 5389 699 705 10.1126/science.282.5389.699 9784122

[B14] Primig M Williams RM Winzeler EA Tevzadze GG Conway AR Hwang SY Davis RW Esposito RE The core meiotic transcriptome in budding yeasts Nat Genet 2000 26 4 415 423 10.1038/82539 11101837

[B15] Friedlander G Joseph-Strauss D Carmi M Zenvirth D Simchen G Barkai N Modulation of the transcription regulatory program in yeast cells committed to sporulation Genome Biology 2006 7 3 R20 10.1186/gb-2006-7-3-r20 16542486 PMC1557749

[B16] Deutschbauer AM Williams RM Chu AM Davis RW Parallel phenotypic analysis of sporulation and postgermination growth in Saccharomycescerevisiae Proceedings of the National Academy of Sciences of the United States of America 2002 99 24 15530 15535 10.1073/pnas.202604399 12432101 PMC137751

[B17] Saccharomyces Genome Database http://www.yeastgenome.org/

[B18] Li F Long T Lu Y Ouyang Q Tang C The yeast cell-cycle network is robustly designed Proceedings of the National Academy of Sciences of the United States of America 2004 101 14 4781 4786 10.1073/pnas.0305937101 15037758 PMC387325

[B19] Wang W Cherry JM Nochomovitz Y Jolly E Botstein D Li H Inference of combinatorial regulation in yeast transcriptional networks: A case study of sporulation Proceedings of the National Academy of Sciences 2005 102 6 1998 2003 10.1073/pnas.0405537102 PMC548531 15684073

[B20] Guttmann-Raviv N Martin S Kassir Y Ime2, a Meiosis-Specific Kinase in Yeast, Is Required for Destabilization of Its Transcriptional Activator, Ime1 Mol Cell Biol 2002 22 7 2047 2056 10.1128/MCB.22.7.2047-2056.2002 11884593 PMC133691

[B21] Suka N Carmen AA Rundlett SE Grunstein M The regulation of gene activity by histones and the histone deacetylase RPD3 Cold Spring Harb Symp Quant Biol 1998 63 391 399 10.1101/sqb.1998.63.391 10384304

[B22] Gershenson C Classification of Random Boolean Networks Artificial Life VIII: Proceedings of the Eight International Conference on Artificial Life: 2002; Sydney, Australia 2002 MIT Press 1 8

[B23] Kauffman SA Metabolic stability and epigenesis in randomly constructed genetic nets Journal of Theoretical Biology 1969 22 3 437 467 10.1016/0022-5193(69)90015-0 5803332

[B24] Liang S Fuhrman S Somogyi R REVEAL, a general reverse engineering algorithm for inference of genetic network architectures Pacific Symposium on Biocomputing: 1998; Hawaii, United States of America 1998 18 29 9697168

