# Supplementary material for: Prediction of quantitative phenotypes based on genetic networks: a case study in yeast sporulation
Source: BMC Syst Biol. 2010 Sep 10;4:128. doi: 10.1186/1752-0509-4-128 (PMC2944141; doi:10.1186/1752-0509-4-128)
Supplement: Additional file 1 — Supplemental materials. Notes about UMMI and construction of the curated and predicted networks. Supplemental Tables S1-4, S10 and Figures S1-2. [file 1752-0509-4-128-S1.DOC]

**Using UMMI to discover enriched sequence constraints**

We compiled a list of 794 DNA motifs and input them to UMMI. Among the 794 motifs, 51 are known ones from literature, 615 are generated by a computational method AlignACE and extracted from [1] and 128 are generated by another computational method called MODEM developed in our group [2], which utilizes both DNA sequences and genome-wide binding data (ChIP-chip).

The gene expression data [3] was pre-processed. Only the genes with an absolute gene expression ratio ≥1.0 in at least one time point were retained. We obtained 3022 genes for further analysis. UMMI is then applied to find the significantly enriched motif combinations and their sequence constraints (see Methods in main text).

**Notes on the curated and predicted networks in Fig. 1 and Fig. 3**

Ime2 is a protein kinase that phosphorylates Ndt80, which is speculated to activate Ndt80 [4]. However, *in vitro* biochemical analysis showed that Ime2’s phosphorylation is not essential to Ndt80’s affinity for its DNA binding sequences [5]. We have investigated this phosphorylation’s effect in our Boolean network: removal of the edge from Ime2 to Ndt80 does not affect the sporulation percentages or the correlations significantly in both predicted and curated networks (Table S10). We added this edge in our networks just to include the hypothetical physical interactions as well.

The Rim4 and Ime2 AND gate was added in Fig. 1 to reflect the fact that Rim4 acts as an RNA-binding protein which stabilizes the Ime2 mRNA [6, 7]. Without Rim4, Ime2 is a highly unstable protein kinase and has a very short half-life [8].

Table S1 UMMI learned sequence constraints at each of the seven time points for yeast sporulation. The computationally generated motifs are marked in parentheses as “Modem” [2] or “AlignACE” [1].

| *Time point* | *Rules* | *Reliability measure* |
| --- | --- | --- |
| 1 (0h) | Presence of M183 (AlignACE):0.65 | 0.45 |
| 2 (0.5h) | Presence of RRPE:0.35  Distance to TSS of PAC:200, 0.05  Presence of UME1 (Modem):0.05  Presence of UME6 (Modem):0.05  Presence of RAP1:0.05  Presence of UME6:0.35  Orientation of RAP1:R, 0.05  Orientation of FHL1 (Modem):F, 0.05  Presence of FHL1 (Modem):0.05 | 0.92  0.86  0.49  0.27  0.25  0.23  0.18  0.16  0.16 |
| 3 (2h) | Distance to TSS of RRPE:300, 0.35  Distance to TSS of MBP1:300, 0.05  Presence of UME1 (Modem):0.05  Presence of FHL1 (Modem):0.05  Presence of LEU3:0.35  Presence of MBP1:0.05  Distance to TSS of UME1 (Modem):200, 0.05  Presence of RRPE:0.35 | 0.75  0.75  0.67  0.49  0.43  0.25  0.24  0.22 |
| 4 (5h) | Distance to TSS of SUM1:300, 0.05  Presence of UME1 (Modem):0.05  Presence of RAP1:0.05  Presence of UME6 (Modem):0.05  Presence of NDT80 (Modem):0.65  Presence of SFP1 (Modem):0.05 | 1.0  0.75  0.73  0.25  0.20  0.20 |
| 5 (7h) | Presence of RAP1:0.05  Presence of SUM1:0.05  Distance to TSS of NDT80 (Modem):300, 0.35  Presence of NDT80 (Modem):0.65  Presence of UME1 (Modem):0.05  Presence of UME6 (Modem):0.05  Distance to TSS of SUM1:300, 0.05  Presence of SFP1 (Modem):0.05  Distance to TSS of UME1 (Modem):200, 0.05  Presence of UME6:0.35 | 0.71  0.69  0.53  0.43  0.33  0.31  0.31  0.18  0.14  0.14 |
| 6 (9h) | Distance to TSS of UME1 (Modem):200, 0.05  Distance to TSS of NDT80 (Modem):300, 0.65  Distance between NDT80 (Modem) and SUM1:100, (0.65,0.05)  Presence of NDT80 (Modem):0.35  Distance to TSS of SUM1:250, 0.05 | 0.98  0.53  0.51  0.47  0.47 |
| 7 (11.5h) | Distance to TSS of SUM1:250, 0.05  Presence of HSF1:0.35  Distance to TSS of NDT80 (Modem):300, 0.35  Distance to TSS of UME1 (Modem):200, 0.05  Presence of NDT80 (Modem):0.65  Distance to TSS of UME6 (Modem):200, 0.05 | 1.0  0.90  0.88  0.88  0.12  0.12 |

Table S2 Perturbation results of the curated network including experimental Prespo/Spore ratios.

| **Gene Name** | **Computational**  **Prediction** | **Prespo/Spore ratio** |
| --- | --- | --- |
| TUP1 | 0.70 | 0.84 |
| MSN2 | 1.09 | 1.32 |
| RIM15 | 1.12 | 2.32 |
| MSN4 | 1.24 | 1.49 |
| RIM11 | 1.33 | 3.95 |
| UME6 | 1.24 | 1.67 |
| NDT80 | 4.42 | 4.33 |
| SOK2 | 0.89 | 0.67 |
| IME2 | 1.16 | 1.59 |
| RPD3 | 0.62 | 0.80 |
| GCN5 | 1.31 | 1.57 |
| SUM1 | 0.98 | 1.12 |
| IME1 | 2.23 | 2.97 |
| CDC28 | 0.94 | 0.85 |
| RIM4 | 1.21 | 5.22 |
| MIG1 | 0.98 | 0.84 |
| CLB1 | 0.97 | 1.15 |
| RAS2 | 0.97 | 1.03 |
| CLN2 | 0.93 | 0.81 |
| RME1 | 0.88 | 0.77 |
| CDC25 | 0.97 | 0.78 |
| CYR1 | 0.92 | 0.72 |

Table S3 Perturbation results of the predicted network including experimental Prespo/Spore ratios.

| **Gene Name** | **Computational**  **Prediction** | **Prespo/Spore ratio** |
| --- | --- | --- |
| IME1 | 1.20 | 2.97 |
| SUM1 | 0.89 | 1.12 |
| UME6 | 1.20 | 1.67 |
| IME2 | 1.44 | 1.59 |
| NDT80 | 3.60 | 4.33 |

Table S4 Perturbation results of the self-activation of the meiotic activators.

| **Perturbation** | **Computational**  **Prediction** |
| --- | --- |
| Rim11 | 1.12 |
| Rim15 | 1.08 |
| Rim11, Rim15 | 1.18 |
| Ime1, Ime2, Ndt80 | 1.12 |
| Ime1, Ime2, Ndt80, Rim11, Rim15 | 1.27 |

Table S10 Impact of the edge from Ime2 to Ndt80. Results are shown for predicted and curated networks before and after the removal of the Ime2 to Ndt80 edge. P-values are shown in parentheses.

|  | Predicted | | Curated | |
| --- | --- | --- | --- | --- |
|  | Before | After | Before | After |
| Sporulation percentage | 0.73 | 0.66 | 0.61 | 0.56 |
| Pearson correlation | 0.87 (0.058) | 0.89 (0.043) | 0.62 ($1.9\times10^{-3}$) | 0.63 ($1.8\times10^{-3}$) |
| Spearman rank correlation | 0.67 (0.27) | 0.67 (0.27) | 0.89 ($1.0\times10^{-6}$) | 0.89 ($1.0\times10^{-6}$) |

Fig. S1 Histogram of the computationally predicted effects of gene double deletion for sporulation specific regulators.

Sporulation efficient

Sporulation neutral

Middle Sporulation deficient

High Sporulation deficient


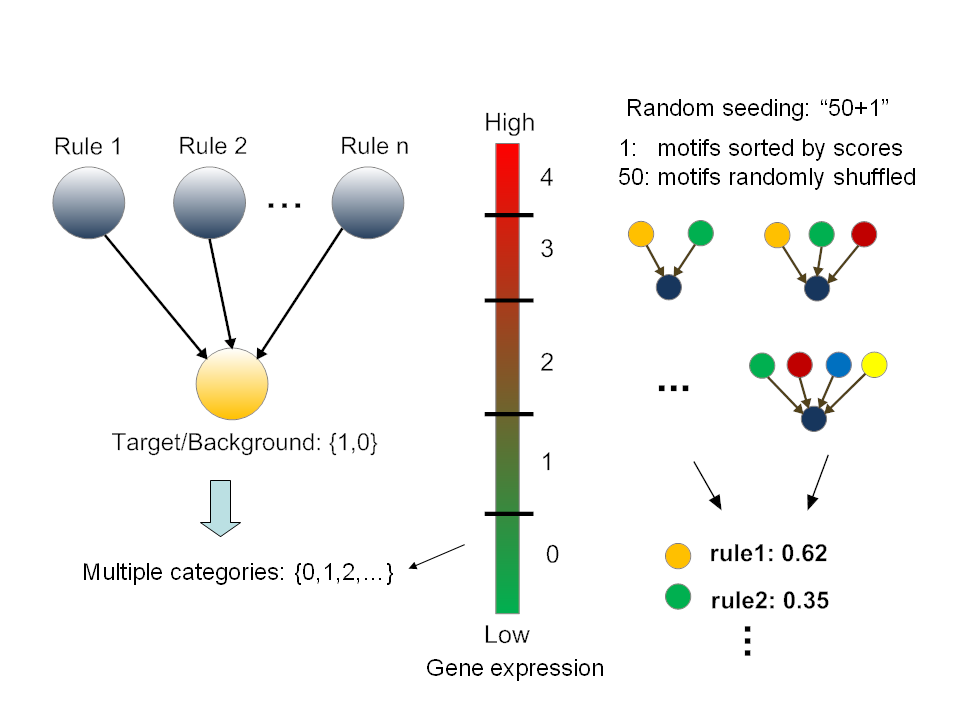


Fig. S2 UMMI: Ubiquitous Model selector for Motif Interactions. UMMI allows a child node to have more than two categories so that a single gene expression microarray dataset can be used as input; UMMI also allows random seeding to find transcriptional regulatory rules with occurrence scores (See main text for description of the method).

Reference

1. Beer M, Tavazoie S: **Predicting gene expression from sequence**. *Cell* 2004, **117**:185-198.

2. Wang W, Cherry JM, Nochomovitz Y, Jolly E, Botstein D, Li H: **Inference of combinatorial regulation in yeast transcriptional networks: A case study of sporulation**. *Proceedings of the National Academy of Sciences* 2005, **102**(6):1998-2003.

3. Chu S, DeRisi J, Eisen M, Mulholland J, Botstein D, Brown PO, Herskowitz I: **The Transcriptional Program of Sporulation in Budding Yeast**. *Science* 1998, **282**(5389):699-705.

4. Sopko R, Raithatha S, Stuart D: **Phosphorylation and maximal activity of Saccharomyces cerevisiae meiosis-specific transcription factor Ndt80 is dependent on Ime2**. *Mol Cell Biol* 2002, **22**(20):7024-7040.

5. Sopko R, Stuart DT: **Purification and characterization of the DNA binding domain of Saccharomyces cerevisiae meiosis-specific transcription factor Ndt80**. *Protein Expr Purif* 2004, **33**(1):134-144.

6. Deng C, Saunders WS: **RIM4 encodes a meiotic activator required for early events of meiosis in Saccharomyces cerevisiae**. *Mol Genet Genomics* 2001, **266**(3):497-504.

7. Soushko M, Mitchell AP: **An RNA-binding protein homologue that promotes sporulation-specific gene expression in Saccharomyces cerevisiae**. *Yeast* 2000, **16**(7):631-639.

8. Guttmann-Raviv N, Martin S, Kassir Y: **Ime2, a Meiosis-Specific Kinase in Yeast, Is Required for Destabilization of Its Transcriptional Activator, Ime1**. *Mol Cell Biol* 2002, **22**(7):2047-2056.
